# A Kiss of Deep Homology: Partial Convergence in the Genomic Basis of Hypertrophied Lips in Cichlid Fish and Human Cleft Lip

**DOI:** 10.1093/gbe/evad072

**Published:** 2023-05-04

**Authors:** Paul Masonick, Axel Meyer, Christopher Darrin Hulsey

**Affiliations:** Department of Biology, University of Konstanz, Germany; Department of Biology, University of Konstanz, Germany; Department of Biology, University of Konstanz, Germany; School of Biology and Environmental Science, University College Dublin, Ireland

**Keywords:** genome resequencing, animal model, human birth defects, evolutionary medicine

## Abstract

The genomic loci generating both adaptive and maladaptive variation could be surprisingly predictable in deeply homologous vertebrate structures like the lips. Variation in highly conserved vertebrate traits such as the jaws and teeth in organisms as evolutionarily disparate as teleost fishes and mammals is known to be structured by the same genes. Likewise, hypertrophied lips that have evolved repeatedly in Neotropical and African cichlid fish lineages could share unexpectedly similar genetic bases themselves and even provide surprising insight into the loci underlying human craniofacial anomalies. To isolate the genomic regions underlying adaptive divergence in hypertrophied lips, we first employed genome-wide associations (GWAs) in several species of African cichlids from Lake Malawi. Then, we tested if these GWA regions were shared through hybridization with another Lake Malawi cichlid lineage that has evolved hypertrophied lips seemingly in parallel. Overall, introgression among hypertrophied lip lineages appeared limited. Among our Malawi GWA regions, one contained the gene *kcnj2* that has been implicated in the convergently evolved hypertrophied lips in Central American Midas cichlids that diverged from the Malawi radiation over 50 million years ago. The Malawi hypertrophied lip GWA regions also contained several additional genes that cause human lip–associated birth defects. Cichlid fishes are becoming prominent examples of replicated genomic architecture underlying trait convergence and are increasingly providing insight into human craniofacial anomalies such as a cleft lip.

**Significance**

Cichlid fishes provide textbook examples of convergent evolution. Their trophic diversity is especially remarkable in that phenotypes like hypertrophied lips have evolved numerous times across disparate adaptive radiations. In this study, we found that the genomic regions underlying adaptive divergence in African Lake Malawi cichlids contained genes that have also been implicated as governing the genomic basis of lip size in a distantly related Neotropical cichlid lineage. Further, several identified candidate loci in Lake Malawi cichlids have also been connected with lip-associated birth defects in humans. Our study suggests that certain ancient genetic pathways in vertebrates may be repeatedly recruited to produce similar lip phenotypes even across vast evolutionary distances.

## Introduction

The genetic basis underlying similar adaptations might be surprisingly conserved and redeployed convergently over vast evolutionary timeframes. Even among highly divergent lineages, both adaptive and maladaptive variation in deeply homologous structures could arise through predictable modifications of the same conserved genomic elements (Elmer and Meyer 2011; Van Otterloo et al. 2016). Convergent adaptations have long provided evidence for the potential of nonrandom phenotypic evolution (Losos et al. 1998; Stiassny and Meyer 1999). It is also becoming increasingly clear that the loci underlying human birth defects like cleft lip and other craniofacial anomalies could reflect a surprising predictability in the genotype to phenotype map guiding vertebrate evolution (Albertson et al. 2009; Van Otterloo et al. 2016). However, although many of the loci responsible for the formation of characteristic vertebrate traits such as jaws, teeth, and lips likely arose in deep time in the common ancestors of teleost fish and humans, the conserved role of particular loci in shaping convergent phenotypes is only now being resolved (Hulsey et al. 2005; Fraser et al. 2009; Hulsey et al. 2010, 2017; Kratochwil et al. 2018; Kautt et al. 2020). But, across the vertebrate tree of life, many groups such as Darwin's finches (Grant and Grant 2008), Caribbean *Anolis* lizards (Losos et al. 1998), and stickleback fishes (Rundle et al. 2000; Marques et al. 2017) have all evolved specific traits repeatedly. Among adaptively radiating lineages, cichlid fishes likely offer the best opportunity to examine the genomic basis of convergent phenotypes over both recent as well as much older evolutionary timescales (Kocher et al. 1993; Meyer 1993; Stiassny and Meyer 1999; Salzburger et al. 2005; Salzburger 2009; Elmer and Meyer 2011; Muschick et al. 2012; [Bibr evad072-B45][Bibr evad072-B52]; Hulsey et al. 2019; Ronco et al. 2021). To identify the comparative genomic basis of a phenotype that has convergently evolved in several cichlid fish adaptive radiations and that could have implications for human craniofacial anomalies, we used a whole GWA approach in Lake Malawi cichlids to delineate the genomic regions underlying the repeated evolution of hypertrophied lips.

Cichlids are known for their convergence in trophic morphology, and hypertrophied lips are among the more externally obvious of these frequently evolved phenotypes (Burress et al. 2013; Colombo et al. 2013; Manousaki et al. 2013; Henning and Meyer 2014; Baumgarten et al. 2015; Henning et al. 2017). Hypertrophied lips have evolved independently in cichlids inhabiting the Nicaraguan crater lakes, South American rivers, as well as repeatedly in the species flocks of African Lakes Tanganyika, Victoria, and Malawi (Fryer and Iles 1972; Burress et al. 2013; Hulsey et al. 2018; Masonick et al. 2022). These hypertrophied lips are an adaptation for foraging in rocky substrates where they aid in sucking invertebrate prey from narrow crevices (Ribbink et al. 1983; Baumgarten et al. 2015), buffer stress during repeated contact with abrasive substrates (Fryer and Iles 1972; Greenwood 1974), and/or facilitate prey detection by providing an enlarged area for taste receptors (Oliver and Arnegard 2010). As well as being trophically adaptive, lips may also play a role in sexual selection (Machado-Schiaffino et al. 2014, 2017). However, considerable plasticity in lip size has also been observed and could indicate that this trait might not always have a clear genetic basis (Machado-Schiaffino et al. 2014, 2017). The important ecological and adaptive consequences as well as the clearly independent origins based on firmly established phylogenetic relationships of hypertrophied lips make them an ideal trait for studying the genomic basis of convergence.

**Fig. 1.**
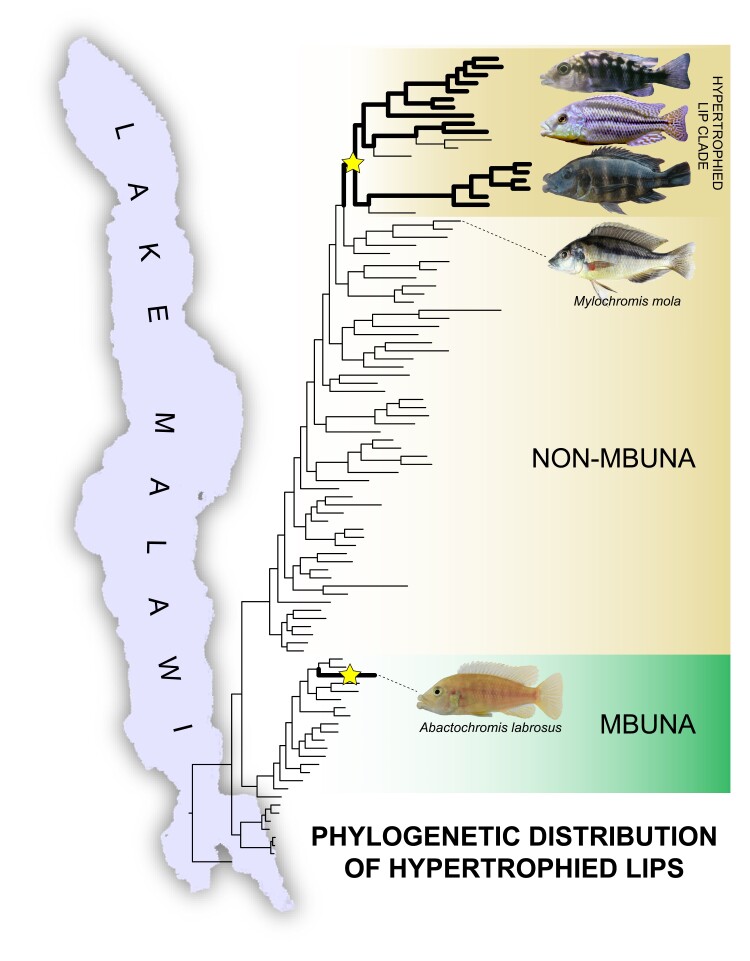
Phylogenetic distribution of the hypertrophied lip phenotype in African Lake Malawi cichlids. Tree modified from Masonick et al. (2022) with hypertrophied lip lineages denoted by thickened branches; this phenotype likely evolved at least twice among the Lake Malawi cichlid fauna (stars). Hypertrophied lips appear to have evolved once in the primarily algae-scraping and rock-dwelling mbuna (*A. labrosus*) and another time among the largely sand-dwelling non-mbuna. Identifying the genomic basis of this phenotype will illuminate whether adaptive introgression has led to the peculiar phylogenetic distribution of this phenotype within Malawi and clarify whether aspects of the genomic basis of this trait are convergently shared with distantly related hypertrophied lip cichlids in the Neotropics as well as human craniofacial and lip anomalies. The phylogenetic position of *M. mola*, a thin-lipped species also included in our data set for GWA, is indicated.

For distinctive cichlid traits such as hypertrophied lips, we can employ genome-wide data to evaluate whether these traits evolved independently, have been retained as ancient polymorphisms, reappeared due to hybridization, or likely arose via the recruitment of novel genomic loci (Elmer et al. 2010; Kautt et al. 2020). The opportunity for hypertrophied lips to have arisen repeatedly and provide a model for recent convergent evolution in Lake Malawi is considerable (Danley and Kocher 2001; Genner and Turner 2005; Konings 2007). Genome-level phylogenetic analyses indicate that hypertrophied lips likely evolved at least twice within the Lake Malawi cichlid adaptive radiation (Masonick et al. 2022) where hypertrophied lips are present both in a lineage containing several species in the largely sand-dwelling non-mbuna and arose independently in the species *Abactochromis labrosus* that is nested within the primarily algae-scraping and rock-dwelling mbuna (fig. 1) (Fryer and Iles 1972; Arnegard and Snoeks 2001; Snoeks 2004; Genner and Turner 2005; Konings 2007; Hulsey et al. 2018; Malinsky et al. 2018; Masonick et al. 2022). Based on these evolutionary relationships, we can now identify closely related Malawi species that do, and do not, exhibit hypertrophied lips for use in GWA studies of the hypertrophied lip phenotype. Once genomic regions are identified, we can subsequently evaluate whether divergence in the same genomic regions is shared in the evolutionarily independent lineages exhibiting exaggerated or aberrant lip phenotypes.

Despite previous phylogenetic inferences that hypertrophied lips seem to have arisen repeatedly in Lake Malawi, introgression of only select regions of the genome could blur the evolutionary independence of these “convergent” phenotypes (Stern 2013). Given that many lineages of Malawi cichlids can be hybridized (Stauffer et al. 1996; Streelman et al. 2004; Mims et al. 2010; Joyce et al. 2011; Scherz et al. 2022) and the entire radiation is only around 2 million years old (Meyer et al. 1990; 1994; Danley and Kocher 2001), interspecific gene flow could play a large role in the putative independent evolution of a trait such as hypertrophied lips. If there were signatures of allele sharing, whether as a result of introgression or even the retention of ancestral polymorphism across the entirety of the Malawi radiation (Moran and Kornfield 1993), this can now be detected in genome-enabled groups like the Malawi cichlids (Conte et al. 2019). Once the genomic regions underlying hypertrophied lips have been identified in one lineage, we could then scan the genome of other putatively independently derived lineages for introgression of those regions underlying lip divergence (Malinsky et al. 2021). If the genetic basis of the hypertrophied lip phenotype was shared among disparate phylogenetic components of the Lake Malawi cichlids, this would provide evidence that “adaptive” introgression could have led to the within lake convergence of hypertrophied lips (Masonick et al. 2022). Alternatively, if we do not find evidence of excess allele sharing in genomic regions associated with hypertrophied lips, this would bolster previous inferences that multiple lineages of Lake Malawi cichlids evolved hypertrophied lips independently.

Because of the distinctiveness of hypertrophied lips, the genomic basis of this repeatedly evolving novelty has been investigated extensively in multiple cichlid radiations (Kautt et al. 2020; Masonick et al. 2022). Transcriptome studies of cichlids from both the Neotropics and Africa identified several differentially expressed genes in species with and without hypertrophied lips (Colombo et al. 2013; Manousaki et al. 2013). However, transcriptomic analyses often suffer from numerous false positives that can obfuscate the number of genetic differences contributing to a trait (Finotello and Di Camillo 2015). Additionally, transcriptomics might only highlight genes downstream of a few rarely expressed master regulators whose genomic divergence actually dictates the presence of this trait (Dowle et al. 2020). Quantitative trait locus (QTL) mapping has also been used to make inferences concerning the identity and number of genomic regions underlying hypertrophied lips in Lake Victoria cichlids (Henning et al. 2017). However, QTLs generally only demarcate broad regions of the genome underlying trait divergence due to the reduced recombination observable in only a few generations of laboratory raised hybrids (Henning et al. 2017). QTLs might also miss crucial loci if they were absent or show segregation distortion in hybrid mapping populations (Henning and Meyer 2014). The ability to sequence whole genomes even in non-model systems like cichlids made it feasible through GWA studies to more precisely delineate the genomic loci underlying phenotypes like hypertrophied lips (Kautt et al. 2020). Replication of GWA studies in evolutionarily independent lineages and/or within the context of previous transcriptomic and QTL results could provide exceptional insights into how robust and generalizable the genotype to phenotype maps in convergent lineages of cichlids are for a trait like hypertrophied lips.

Knowing whether fish lineages that evolved within the same lake or on different continents employ comparable genomic changes to produce highly similar phenotypes has implications beyond adaptive radiations of cichlids. What is increasingly clear with respect to craniofacial divergence is that many genes might play roles in not only adaptive but also potentially maladaptive phenotypic divergence in lineages as evolutionarily disparate as cichlid fishes and humans (Albertson et al. 2009; Wolf et al. 2015; Orr et al. 2016; Van Otterloo et al. 2016; Usui and Tokita 2018). For instance, a recent GWA study of hypertrophied lips in the Central American Midas cichlids found that a gene responsible for cleft lip in humans, *kcnj2*, fell within one of only two associated genomic regions that underlie hypertrophied lips (Kautt et al. 2020). However, the genetic basis of many human craniofacial anomalies, even with whole genome sequencing, can often only be narrowed down to relatively broad candidate regions that contain several loci (Usui and Tokita 2018). Therefore, genomic investigations of hypertrophied lips in additional cichlid lineages could strengthen inferences concerning the importance of particular loci to vertebrate craniofacial divergence in general and in human birth defects such as cleft lip in particular (Diewert and Lozanoff 2002; Albertson et al. 2009; Wolf et al. 2015; Van Otterloo et al. 2016). The genotype to phenotype map might be surprisingly similar for craniofacial traits that are adaptive in one group of vertebrates and maladaptive in another.

In this study, we employed GWA to explore the comparative genomic basis of lip phenotypes at several evolutionary timeframes. We first generated novel whole genome sequences and performed GWA mapping to isolate the genomic basis of hypertrophied lips in a clade of non-mbuna hypertrophied lip species from Lake Malawi. Then, we tested whether introgressive gene flow could explain the hypertrophied lip phenotype in the putatively convergent hypertrophied lip Lake Malawi mbuna species *A. labrosus*. Additionally, through comparisons with other genome level studies, we identified whether the genomic regions implicated in governing the Malawi hypertrophied lip phenotype contain loci underlying convergent lip phenotypes in other cichlid fishes. Finally, we determined whether any loci contained in the genomic regions associated with hypertrophied lips in Malawi cichlids have been found to influence human craniofacial birth defects like cleft lip.

## Results

### GWA Mapping

**Fig. 2.**
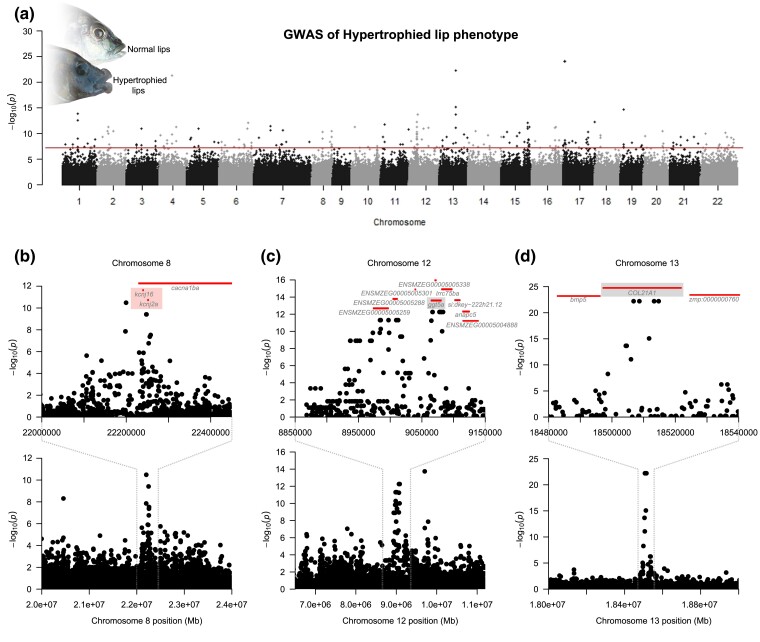
GWA mapping of the hypertrophied lip phenotype in Lake Malawi cichlids. (*a*) Manhattan plot arranged by chromosome number (1–22). Numerous high signals of association (red line denotes 233 SNPs surpassing the threshold of −log_10_(*P*) = 7.301) were detected across the genome. (*b*–*d*) Genomic windows with gene annotations of candidate lip loci on chromosomes 8, 12, and 13 are highlighted. Several genes occurring around identified peaks of high association have been found to be associated with the hypertrophied lip phenotype in other cichlid studies. (*b*) For instance, *kcnj16* and *kcnj2a* are genes encoding for inward rectifier potassium channels and underlie the convergently evolved hypertrophied lip phenotype in Midas cichlids (Kautt et al. 2020). (*c*) The genes *ggt5a* and *lrrc75ba* and several other protein-coding genes are dispersed around a major peak of association located on chromosome 12. (*d*) In addition to these, *col21a1*, a gene implicated in the development of cleft lips in humans, overlaps with one of the strongest regions of genomic association identified. Supplementary table S5, Supplementary Material online, provides a list of annotated genes located within 50 kbp of these highest-associated SNPs.

Our GWA study was based on 1,737,749 single nucleotide polymorphisms (SNPs) derived from whole-genome resequencing of 39 Lake Malawi cichlid species (supplementary table S1, Supplementary Material online). We found high genomic associations for the hypertrophied lip phenotype at many loci across the genome (fig. 2). Nearly every chromosome (21 of 22) carried SNPs (233 in total) that displayed high association (i.e., surpassing a genome-wide significance threshold of −log_10_(*P*) = 7.301 [i.e., *P* < 5e^−8^]) with the trait (supplementary table S2, Supplementary Material online). The 100-kbp windows centered around each significant SNP contained 407 genes, 276 annotated genes that have mammalian or zebrafish homologs and 131 annotated genes without clear homologs in other vertebrate models.

### Analysis of Gene Flow

**Fig. 3.**
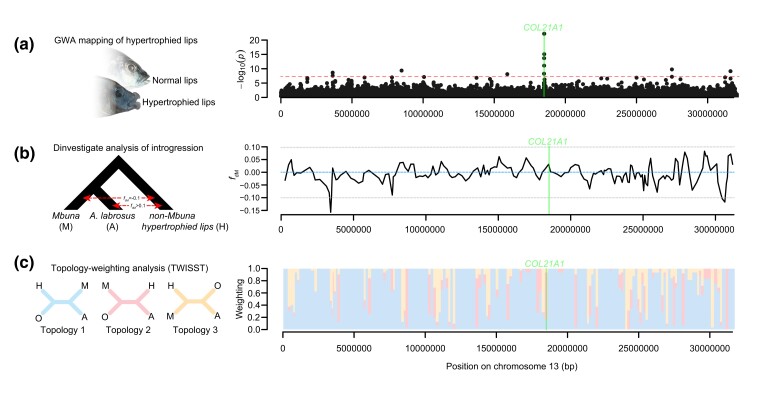
Evaluation of introgressive hybridization of hypertrophied lip–associated SNPs in Lake Malawi cichlids along chromosome 13. The genomic position of the highly associated hypertrophied lip gene *col21a1* is indicated by the green bar in graphs *a*–*c*. (*a*) We investigated whether GWA regions identified in the non-mbuna hypertrophied lip clade showed high associations within the lip size. Highly associated SNPs (*P* < 5e^−8^) are distinguished by the red line. (*b*) Genomic regions were examined using Dinvestigate to pinpoint regions showing higher than expected levels of introgression (high *f*_dM_ statistic calculated in sliding windows of 250 SNPs) in *A. labrosus* compared with other mbuna. The blue line denotes no introgression (*f*_dM_ = 0.0) whereas values strongly departing from 0.0 are more consistent with gene flow between the non-mbuna hypertrophied lip species and either *A. labrosus* (*f*_dM_ > 0.1) or the mbuna sans *A. labrosus* (*f*_dM_ < −0.1). (*c*) Additionally, we used a topology-weighting analysis (TWISST) to investigate whether the hypertrophied lip mbuna and non-mbuna were more likely to be monophyletic at putative GWA regions than expected by chance. Topology 1 (blue) reflects the accepted species tree (the mbuna (M) and *A. labrosus* (A) are monophyletic), Topology 2 (red) indicates shared phylogenetic signal between *A. labrosus* and non-mbuna haplochromines with hypertrophied lips (H), and Topology 3 (yellow) represents shared history between the mbuna (sans *A. labrosus*) and non-mbuna hypertrophied lip species. The non-Lake Malawi *H. bloyeti* was used as an outgroup (O). The raw topological frequency weighting was estimated across nonoverlapping windows of 250 SNPs spanning chromosome 13 and is plotted here. In general, high GWA regions in the hypertrophied lip non-mbuna (fig. 2*a*) do not show clear patterns of introgression into the Malawi mbuna hypertrophied lip species *A. labrosus* based on the Dinvestigate results; however, *col21a1* is nested within a region that is enriched for Topology 2 according on the TWISST analysis.

Gene flow analyses revealed numerous potentially introgressed genomic regions from the non-mbuna hypertrophied lip species. Using the Dinvestigate tool in the program Dsuite (Malinsky et al. 2021), we did find some patterns of genomic introgression between the mbuna hypertrophied lip species *A. labrosus* and the non-mbuna hypertrophied lip species as well as loci that appear to have introgressed between other mbuna cichlids (sans *A. labrosus*) and the hypertrophied lip non-mbuna (supplementary fig. S1, Supplementary Material online and supplementary table S3*a*, Supplementary Material online). However, large spans of the *A. labrosus* genome yielded *f*_dM_ scores that are indicative of no introgression (i.e., *f*_dM_ = ∼0.0). Altogether, the genome-wide average *f*_dM_ score was 0.001529, and the average taken across Dinvestigate windows containing significant GWA SNPs was −0.000433 (supplementary table S3*b*, Supplementary Material online). Only in two instances do significant GWA SNPs coincide with Dinvestigate windows that resulted in *f*_dM_ scores that show strong signal of introgression (i.e., *f*_dM_ > 0.1), once for a window spanning 28,225,540–28,557,187 on chromosome 6 and again on chromosome 11 between base pair positions 22,438,916 and 22,701,993. Protein-coding genes located within these two putatively introgressed regions of hypertrophied lip taxa include differentially expressed in normal cells and neoplasia domain-containing protein 1C (si:dkey-76b14.2), tubulin beta-4B chain (*zgc:65894*), and glucagon receptor b (*gcgrb*) on chromosome 6 and persulfide dioxygenase (*ethe1*), dopamine receptor D2 like (*drd2l*), X-ray repair cross complementing 1 (*xrcc1*), and a myelin-associated glycoprotein on chromosome 11. However, despite several portions of the genome carrying signatures of potential gene flow among the mbuna *A. labrosus* and non-mbuna hypertrophied lip species, we found little overlap with these regions and those associated with the putative genomic basis of the hypertrophied lip phenotype implicated in the GWA analysis (fig. 3*a*–*c*). Although Dinvestigate scores corresponding to the SNP windows spanning the *col21A1* locus were positive (mean *f*_dM_ = 0.015091, fig. 3*b*), the estimated *f*_dM_ values were well below the 0.1 threshold used to diagnose putative introgression between *A. labrosus* and the non-mbuna hypertrophied lip species.

The phylogenetic weighting approach (topology weighting by iterative sampling of subtrees [TWISST]) employed here revealed largely similar patterns to the Dinvestigate analyses of gene flow (supplementary fig. S2, Supplementary Material online and supplementary table S4, Supplementary Material online). Although scanning windows of × 250 SNPs across the genome with TWISST recovered substantial support for the general species tree topology reported by Masonick et al. (2022) (i.e., that *A. labrosus* + the mbuna comprise a clade), we also found numerous relatively narrow genomic regions that exhibited elevated support for topologies incongruent with that of the species tree. Of the 4,039 nonoverlapping total TWISST windows each spanning 250 SNPs, 1,453 (35.97%) showed nonzero weightings for the alternative topology grouping *A. labrosus* with the non-mbuna hypertrophied lip haplochromines (supplementary table S4*a*, Supplementary Material online; see Topology 2 in fig. 3*c*). Of these, 574 (14.21% of total) windows exhibited scores where this specific alternative topology received a greater weighting than that of the species tree. Significant GWA SNPs (×29 = 12.45% of total) overlapped with only 14 of these particular TWISST windows scattered across the genome (supplementary table S4*b*, Supplementary Material online). Candidate genes coinciding with TWISST windows exhibiting phylogenetic signal that link *A. labrosus* with other hypertrophied lip species are indicated in supplementary table S2, Supplementary Material online. Some SNPs occurring within genes implicated in human cleft lip and other congenital craniofacial anomalies, such as *col21A1*, overlap with TWISST windows that exhibit an elevated signal for this alternative topology (fig. 3*c* and supplementary table S4*b*, Supplementary Material online). Either ancestral gene flow and/or incomplete lineage sorting could have contributed to this signal, but TWISST does not explicitly distinguish between the two.

Taking both the Dinvestigate and TWISST analyses into account with the positions of GWA SNPs, we find scant evidence that adaptive introgression has occurred between the divergent hypertrophied lip taxa investigated. Given this and the phenotype's seemingly complex, polygenic nature, the hypothesis that multiple lineages of Lake Malawi cichlids have evolved hypertrophied lips independently remains valid.

### Genomic Convergence with Other Similar Vertebrate Phenotypes

Based on genomic studies in other cichlids and genes implicated in human craniofacial anomalies, we subsequently identified numerous candidate genes for lip phenotypes that are located upstream and downstream of the 50-kbp windows bracketing significantly associated SNPs (supplementary table S5, Supplementary Material online). Several genes in the vicinity of these peaks of high-association have been linked to the presence of hypertrophied lips in prior cichlid studies. For instance, on chromosome 8, we found highly-associated SNPs neighboring two inward rectifier potassium channels (*kcnj16* and *kcnj2a*) (fig. 2*b*), which were previously associated with the hypertrophied lip phenotype in Midas cichlids native to Central America (Kautt et al. 2020). In contrast, DAB adaptor protein 2 (*dab2*) and prostaglandin e receptor 4 (*ptger4*), which were also associated with this phenotype in Midas cichlids (Kautt et al. 2020), were not found to occur near any of the major peaks of association revealed in our analysis on Malawi cichlids (supplementary table S5, Supplementary Material online). Additionally, we identified a number of genes within these windows that have been implicated in human cleft lip and other congenital craniofacial anomalies. These include genes such as gamma-glutamyltransferase 5a (*ggt5a*) on chromosome 12, collagen type XXI alpha 1 chain (*col21a1*) on chromosome 13, and pleckstrin homology domain-containing family A member 5 (*plekha5*) on chromosome 17 (table 1). Whereas the SNP showing the highest association with the phenotype (SNP# 1294941, *P* = 1.00e^−24^) on chromosome 17 (bp position 2,003,131) does not occur within any gene annotated in the *Maylandia zebra* reference genome, it is found near (within 50 kbp of) the gene phosphorylated adaptor for RNA export (*phax*), peptidase mitochondrial processing subunit beta (*pmpcb*), and DnaJ (Hsp40) homolog subfamily C member 2 (*dnajc2*).

**Table 1:** Genes Overlapping Our Significant GWAS SNPs in Malawi Cichlids That Have Been Connected with Craniofacial Anomalies in Humans and/or Mice

Gene name (Malawi cichlids)	Protein/transcription factor	Name (human/mouse)	Reference
*dusp22a*	Dual specificity protein phosphatase 22-A	*dusp22*	Chen et al. 2013
*smarce1*	SWI/SNF-related matrix-associated actin-dependent regulator of chromatin subfamily E member 1	*smarce1*	Kosho et al. 2014
*igf2b*	Insulin-like growth factor 2	*igf2*	Eggenschwiler et al. 1997
*mamdc2a*	MAM domain containing 2	*mamdc2*	Kuniba et al. 2009
*kcnj2*	Inward rectifier potassium channel 16	*kir2.1* (same gene)	Belus et al. 2018
*VPS13B*	Vacuolar protein sorting 13 homolog B	*VPS13B*	Dehghan et al. 2021
*ggt5a*	Gamma-glutamyl transferase 5	*ggt5*	Pinchefsky et al. 2017
*ptch1*	patched 1	*PTCH1*	Carter et al. 2010
*col21A1*	Collagen type XXI alpha 1 chain	*col21A1*	Mohamad Shah et al. 2019
*CBL*	Cbl proto-oncogene	*CBL*	Martinelli et al. 2020
*dnmt3aa*	DNA (cytosine-5)-methyltransferase 3A	*dnmt3a*	Zhang et al. 2018
*TGFB3*	Transforming growth factor beta-3	*tgfb3*	Kim et al. 2003
*hspa14*	Heat shock protein family A (Hsp70) member 14	*hspa14*	Zhu et al. 2013
*phax*	Phosphorylated adaptor for RNA export	*phax*	Ansari et al. 2014
*plekha5*	Pleckstrin homology domain-containing family A member 5	*plekha5*	Cox et al. 2018
*porcn*	Porcupine O-acyltransferase	*porcn*	Wright et al. 2016
*SATB2*	DNA-binding protein SATB2	*SATB2*	FitzPatrick et al. 2003

## Discussion

The genomic basis of Lake Malawi cichlid hypertrophied lips provides insight into convergence in the genotype to phenotype map over multiple evolutionary timescales. First, GWA indicates that several genomic regions in Lake Malawi non-mbuna cichlids underlie the presence of hypertrophied lips. We also found minimal evidence that these genomic regions have adaptively introgressed into the hypertrophied lip mbuna *A. labrosus*. Further, a large number of the genomic regions associated with hypertrophied lips in Lake Malawi contain loci that might be responsible for not only the presence of hypertrophied lips in other cichlid lineages but also could underlie human craniofacial abnormalities like cleft lip.

There were several genomic regions found to be associated with the presence of hypertrophied lips in the Lake Malawi cichlids (fig. 2), and many of these could convergently underlie the evolution of lip phenotypes in other vertebrates. The region with one of the strongest associations contained the gene collagen type XXI alpha 1 chain (*col21A1*). As this gene is known to influence cleft lip in humans (Mohamad Shah et al. 2019), it provides an excellent candidate locus for hypertrophied lip formation in cichlids. Most chromosomes were also found to exhibit peaks of association with lip size in Malawi, and a large number of these also contain loci linked to cleft lip in humans. Some of the most noteworthy of these loci bracketed by SNPs in GWA peaks include genes like *dusp22*, *tgfb3*, *vps13b*, *satb2*, and *ptch1* that have all been connected to human craniofacial anomalies and birth defects (table 1). Many of these peaks also fall within regions that have been identified through QTL analyses of hypertrophied lips in Lake Victoria cichlids (Henning et al. 2017). The recent divergence of the cichlids inhabiting Lake Malawi and Lake Victoria make it likely that many of the same genomic loci could influence an adaptive trait like hypertrophied lips in both lakes (Meyer 1993; Salzburger and Meyer 2004; Hulsey et al. 2017; Kratochwil et al. 2018). The gene *igf2b* is perhaps the most notable gene located in one of our GWA peaks that was also previously recovered in transcriptome studies of hypertrophied lips (Lecaudey et al. 2021). The gene inward rectifier potassium channel 2 (*kcnj2*) was similarly located under one of our Malawi GWA peaks, and it is another locus that is known to cause cleft lip in humans (Kosho et al. 2014). But, perhaps even more surprising, this gene has also been found to be genomically associated with hypertrophied lips in the Midas cichlids native to the Neotropics (Kautt et al. 2020). To our knowledge, this provides not only a first possible common genetic link between repeated cichlid and human lip phenotypes but also the first evidence of cichlid morphological convergence in the Neotropical and African cichlid radiations due to divergence at convergent genomic loci.

**Fig. 4.**
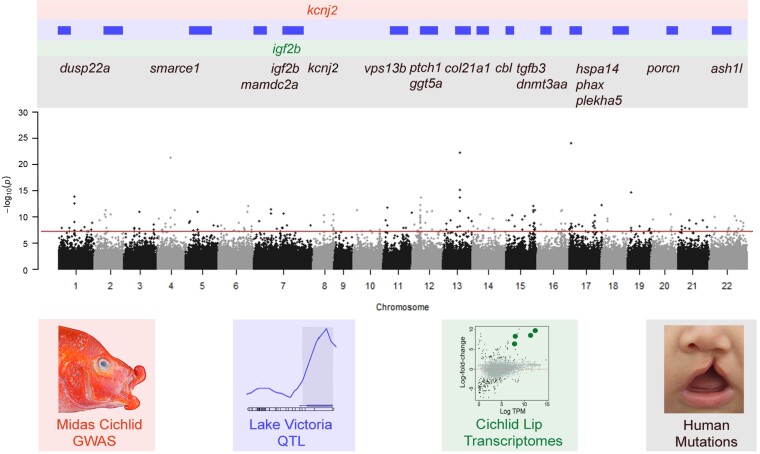
Various hypertrophied lip–associated loci aligned to our chromosome-level GWA results. Previous studies that employed GWA mapping (red) in Midas cichlids (Kautt et al. 2020), QTL analysis (blue) of a hypertrophied lip species from Lake Victoria (Henning et al. 2017), and comparative transcriptomic gene expression (green) of lips in various lineages of cichlids (Colombo et al. 2013; Manousaki et al. 2013; Lecaudey et al. 2021), along with the GWA mapping conducted herein using hypertrophied lip cichlid species from Lake Malawi (Manhattan plot), have identified a number of loci that overlap with genes linked with craniofacial and lip anomalies in humans (black). The Lake Victoria QTL regions (supplementary table S6, Supplementary Material online) are shown primarily to highlight how they demarcate wide intervals on the chromosomes that in some cases could overlap peaks of association reported here for Malawi cichlids. Also, these QTLs, in conjunction with our GWA mapping, are consistent with a polygenic basis to hypertrophied lips in East African cichlids.

Despite a few aspects of the genomic basis of hypertrophied lip phenotypes being shared, the total number of loci responsible for hypertrophied lips in the Neotropical and African cichlid radiations is likely to be fundamentally different. Our GWA results suggest that the presence of hypertrophied lips in Lake Malawi is a polygenic trait (fig. 1). Similar GWA analyses performed in Midas cichlids found hypertrophied lips to be governed by at most two loci (Kautt et al. 2020). Yet, the polygenic basis we recovered for the hypertrophied lip phenotype is consistent with other previous genome level studies in other African adaptive radiations focused on species with hypertrophied lips (fig. 4; Henning et al. 2017; Colombo et al. 2013; Lecaudey et al. 2021). Recent transcriptomic studies in East African cichlids also point to there being many genes that underlie similar craniofacial adaptations (Lecaudey et al. 2021; Duenser et al. 2022). Furthermore, a QTL analysis of hypertrophied lip species in Lake Victoria cichlids that are closely related to those found in Lake Malawi also recovered a highly polygenic basis for this trait (supplementary table S6, Supplementary Material online; Henning et al. 2017). However, the multiple peaks of genomic association we recovered between hypertrophied and normal lip Malawi species must also be evaluated with caution as there are traits other than lips that have phenotypically diverged among the cichlid species examined (Fryer and Iles 1972; Snoeks 2004). Genomic changes underlying these peaks of association could instead influence divergence in traits as disparate as body pigmentation, teeth, and other craniofacial skeletal elements that differ among these species with the commonality of hypertrophied lips (Fraser et al. 2009; Kratochwil et al. 2018; Hulsey et al. 2019). Therefore, more genomic analyses will be necessary to narrow down the number of loci contributing to the genetic basis of the lip size in the Lake Malawi cichlids.

Caution about the contribution of genomic regions to lip divergence is also warranted as the use of multiple species in GWA can produce spurious associations for traits. Extensive fixation of alleles can occur not only because of adaptive divergence but also due to nonadaptive processes such as genetic drift (Buffalo and Coop 2020). Species can often exhibit fixed differences due to random processes following population subdivision or speciation (Kautt et al. 2020). However, unlike most groups of organisms, the speciation events in Malawi cichlids have occurred over exceptionally short timeframes as several hundred cichlid species have arisen within this group in approximately 2 million years (Genner and Turner 2005; Genner et al. 2015). This incredibly fast speciation rate has resulted in the substantial retention of ancestral polymorphism providing genomic null distributions against which phenotype-specific associations can be contrasted (Brawand et al. 2014; Kautt et al. 2020; Scherz et al. 2022). Additionally, introgression might often occur among Malawi species (Mims et al. 2010; Holzman and Hulsey 2017), and this would add to the lack of genome-wide differentiation among species except where trait-specific differences generate clear genomic differentiation. However, differences in sample sizes can increase false negative rates (resulting in associations pertinent to the smaller data set not being discovered) and thus reduce the accuracy of a GWA study (Hong and Park 2012). Even though we essentially treated all hypertrophied lip non-mbuna cichlids in our analysis as one group, some species (e.g., *Placidochromis* “Mbenji fatlip”) were represented by just a single individual. Genetic signal associated with the lip phenotype and unique to these poorly represented species could possibly be obscured by signal from more thoroughly sampled taxa (e.g., *Placidochromis milomo* or *Mylochromis mola*). But, these caveats are not exceptional. All GWA studies are most informative when the results are coupled with additional types of genomic data or confirmed to be important in convergently evolved species with similar phenotypes.

The two phylogenetically independently lineages of Malawi cichlids with hypertrophied lips both co-occur in the same rocky habitats (Ribbink et al. 1983; Masonick et al. 2022). Because many species of Malawi cichlids are known to hybridize in both the laboratory and in the wild (Streelman et al. 2004; Mims et al. 2010; Scherz et al. 2022), the genomic regions underlying the lip phenotype could have readily introgressed from the non-mbuna hypertrophied lip species into the mbuna hypertrophied lip species. Yet, we did not find clear evidence for this type of introgressive hybridization (see figs. 2, S1, and S2, Supplementary Material online and supplementary table S2, Supplementary Material online). Based on comparisons of the genomic regions highly associated with the trait in the non-mbuna, most GWA regions showed limited signs of introgression into the relatively distantly related mbuna *A. labrosus*. Of the 233 highly associated SNPs, 190 (∼81.5%) of these occur in regions that recovered higher support for the species tree than the alternative topologies. Only three (∼1.29%) occur within genomic windows that yielded significant Dinvestigate scores (supplementary table S2, Supplementary Material online). However, the region containing *col21a1* is worth noting. It had one of the highest GWA with hypertrophied lips. Further, the patterns observed from Dinvestigate and TWISST analyses, although not providing clear indication of gene flow, do not discount the possibility that some allele sharing has occurred at this locus (fig. 3). Unfortunately, the limited sample size of *A. labrosus* (*n* = 1) in our genomic data set currently prohibits us from conducting a meaningful GWA. However, future transcriptomic and/or QTL centered analyses in both the rock-dwelling and sand-dwelling hypertrophied lip lineages in Malawi or GWA focused on *A. labrosus* and other mbuna might more clearly delineate the shared genomic underpinnings of the repeated origin of this distinctive lip phenotype.

One of the most fascinating insights of the genomic era is the ever-increasing evidence that the same loci can often be modified repeatedly to produce phenotypically similar traits across deeply divergent clades (Elmer and Meyer 2010). For instance, changes in vertebrate traits like teeth, eyes, and pigmentation often result from the repeated modification of homologous loci (Fraser et al. 2009; Henning and Meyer 2014, Kratochwil et al. 2018). Despite the vast number of base pairs contained in vertebrate genomes, our data suggest that particular genetic pathways are recruited repeatedly to bring about similar phenotypes and convergently evolve adaptations. Even more surprising, this replication occurs both among relatively closely related species as well as across vast evolutionary distances. This conservation has far-reaching implications and could even inform human health. For instance, cleft lip is the most common birth defect in humans and affects over 100,000 newborns worldwide each year (Wilson-Nagrani et al. 2018). Despite extensive biomedical research, the genetic underpinnings of cleft lip remain unclear, and multiple genes have been suggested as potentially underlying this birth defect (Carter et al. 2010; Martinelli et al. 2020). A surprisingly large number of the genes uncovered here in Malawi cichlids that are associated with the hypertrophied lip phenotype are also homologous to the genes implicated in human cleft lip (table 1). This apparent similarity of the genomic basis provides comparative support for the involvement of these genes both in this adaptive cichlid trait as well as in human pathologies (Albertson et al. 2009). Implicating these genes in lip divergence is an especially promising finding because even with the widespread availability of inexpensive whole genome sequencing in humans, the ambiguity often associated with the genetic causes of human pathologies are often difficult to resolve satisfactorily (Wolf et al. 2015). Human craniofacial anomalies and birth defects arising due to mutations in particular loci might often be inherited in a relatively few and often closely related individuals that cannot be experimentally manipulated (Wilson-Nagrani et al. 2018; Martinelli et al. 2020). Therefore, the genomic framework to narrow down whether a certain gene is responsible for a human phenotype is often not tractable (Carter et al. 2010). Additionally, when a trait like a human birth defect is successfully isolated in the genome, genomic linkage often prevents the genetic underpinning of the trait from being unambiguously dissected to a resolution more precise than a given genomic cluster containing several genes (Kang et al. 2010). Genomic scans in nonhuman vertebrates that exhibit novelty in homologous craniofacial elements like the hypertrophied lips in cichlids could provide enormous power to validate whether particular genes underlie human birth defects.

Cichlid fishes are widely known for their convergent phenotypic evolution (Meyer 1993, Stiassny and Meyer 1999). Recently, these examples of repeated adaptive diversification are also becoming tractable models of convergent evolution at the genomic level (Kratochwil et al. 2018; Kautt et al. 2020). This type of partial genomic convergence increasingly provides some of the strongest evidence that evolution is not random or entirely historically contingent. Additionally, as the mechanistic underpinnings of convergent phenotypes are clarified, we are beginning to learn when one might expect the genomic basis of similar phenotypes to be the same and when we should expect them to be completely different (Peichel et al. 2001; Schluter et al. 2004; Elmer and Meyer 2011; Stern 2013; Hulsey et al. 2019). Knowing whether there are a vast or only a limited number of genomic mechanisms that will generate the same phenotypic outcome will become increasingly feasible as we continue to expand the genomic dissections of traits in non-model wild-caught organisms (Hulsey et al. 2005; Wise and Stock 2010). Because they exhibit strong similarities over both more recent as well as over longer timeframes, cichlid fishes offer a powerful system for studying convergence (Hulsey 2009; Henning and Meyer 2014; Brawand et al. 2014). Additionally, because of the conservation of developmental genetic mechanisms across vertebrates, cichlid fishes could increasingly shine light on medically relevant human craniofacial anomalies such as cleft lip. As the genomic basis of replicated vertebrate phenotypes like cichlid hypertrophied lips is investigated, we will continue to discover more unexpected instances of a shared genomic basis to both adaptive and maladaptive divergence in deeply homologous structures.

## Materials and Methods

### Whole-Genome Resequencing, Mapping, and Variant Discovery

To examine GWAs for hypertrophied lips in Lake Malawi, we employed whole-genome resequencing data for 39 Lake Malawi cichlid individuals sampled from eight species: four species that possess hypertrophied lips (*Cheilochromis euchilus*, *Eclectochromis ornatus*, *P. milomo*, and *Placidochromis* “Mbenji fatlip”) and four that do not (*Chilotilapia rhoadesii*, *Hemitaenichromis spilopterus*, *Placidochromis johnstoni*, and *M. mola*) (supplementary table S1, Supplementary Material online). This chosen combination of species was guided by their close phylogenetic affinities, yet their remarkably different lip phenotypes (Masonick et al. 2022). The species *C. rhoadesii*, *H. spilopterus*, and *P. johnstoni* are all nested within a clade containing the four hypertrophied lip species, and *M. mola* is part of this clade's sister group. In addition to leveraging available whole genome sequence data for 24 individuals from these eight species (Malinsky et al. 2018, Masonick et al. 2022; Scherz et al. 2022), we produced genome sequences for 15 additional *M. mola* to boost the sample size of closely related cichlid genomes that differ in the possession of hypertrophied lips.

Prior to sequencing, high-molecular-weight DNA was extracted from fin or muscle tissue using a QIAGEN DNeasy Blood & Tissue Kit although including an RNase A treatment step. DNA integrity was verified on agarose gels and concentrations measured on a QuBit fluorometer. Genomic libraries were prepared with Illumina TruSeq DNA Nano kits targeting insert sizes of 350 bp and then paired-end sequenced (2 × 150 bp) on Illumina HiSeq platforms at the Beijing Genome Institute. Four individuals were pooled per lane aiming for an approximate genome coverage of at least 20× per individual (mean read depth per sample are listed in supplementary table S1, Supplementary Material online).

Following demultiplexing of sequenced libraries, unmapped BAM files were generated from the raw FASTQs with Picard Tools v2.7.1 (FastqToSam) although marking Illumina adapters in the process (MarkIlluminaAdapters). Reads were then converted back to FASTQ format (SamToFastq) and mapped against the 22 chromosome assemblies of the latest version of the high-quality Malawi cichlid *M. zebra* reference genome (GCA_000238955.5: M_zebra_UMD2a of Conte et al. 2019) using bwa -mem v0.7.17 (Li 2013). Metadata stored in the original unmapped BAM files were then added to the aligned BAM files using Picard MergeBamAlignment. Polymerase chain reaction duplicates were annotated with Picard MarkDuplicates.

Prior to this study, we called variants simultaneously for 191 Lake Malawi cichlids (including the 15 *M. mola* samples reported above as well as the hypertrophied lip taxa examined by Masonick et al. (2022)) using freebayes v1.3.1 (Garrison and Marth 2012) although implementing standard default quality filters (data unpublished). Variants of the 39 samples used herein for the GWA analysis were extracted from this initial VCF file using bcftools v1.11 (Li et al. 2009; Danecek et al. 2021). The resulting sub-setted VCF file was then hard-filtered using the vcffilter script from the vcflib package (https://github.com/vcflib/vcflib) (command: -s -f “QUAL > 1 & QUAL / AO > 10 & SAF > 0 & SAR > 0 & RPR > 1 & RPL > 1”). Variants were then normalized and duplicates removed using the tools normalize and uniq of the program vt v0.570.5 (Tan et al. 2015). Lastly, the 39 sample VCF file was filtered with bcftools to produce a data set of biallelic SNPs using the same depth, genotype quality, unique site, and minor allele frequency filtering schemes reported in Masonick et al. (2022). This yielded a VCF file containing 1,737,749 SNPs for the 39 species’ genomes. These SNPs were then imputed and phased with beagle v5.1 (Browning et al. 2018) prior to GWA analysis.

### GWA Mapping

A GWA analysis was performed on the cloud-based easyGWAS platform (Grimm et al. 2017) with the EMMAX algorithm (Kang et al. 2010) and default settings (additive SNP encoding, 0% minor allele frequency filter). To guide this analysis, we used a phenotype file that indicated a hypertrophied lip score (treated here as a binary trait: present = 1 or absent = 0) for each genomic sample. Genes located entirely or partially within 100-kbp windows centered on the SNPs (50-kbp upstream and 50-kbp downstream) occurring above the standard genome-wide *P*-value threshold (*P* < 5e^−8^) (Chen et al. 2021) are reported in supplementary table S5, Supplementary Material online. This genomic window size around significant SNPs was used because such mutations could readily modify cis regulatory elements influencing genes contained in these regions (Stranger et al. 2007). To visualize these results in detail with respect to chromosomes, Manhattan plots were produced with the R package qqman v0.1.8 (Turner 2018).

### Analyses of Gene Flow

Cichlid species in Lake Malawi possessing hypertrophied lips are found not only within the non-mbuna radiation of Lake Malawi cichlids examined above with GWA but also within the phylogenetically distinct mbuna radiation (Masonick et al. 2022). Therefore, we investigated the genomic histories of these two putatively independent origins of hypertrophied lips in Lake Malawi further to determine if introgression may have led to the peculiar phylogenetic distribution of this phenotype (fig. 1). We were particularly interested in whether any genomic regions displaying signal of introgression overlapped with SNPs correlated with the hypertrophied lip trait in our non-mbuna GWA study. To examine this putative introgression, we employed two approaches: 1) analysis of gene flow with sliding window *D*-statistics and 2) phylogenetic topology weighting.

### Sliding Window *D*-Statistics

To locate specific regions exhibiting elevated patterns of introgression among hypertrophied lip Malawi cichlids, we used the Dinvestigate tool in the program Dsuite v0.4 r38 (Malinsky et al. 2021). This program was used to calculate *D*-statistics (also referred to as ABBA–BABA statistics) and related metrics across sliding genomic windows of 250 SNPs spaced across 100 SNP intervals on the master 1,352,537 SNP data set of Masonick et al. 2022. This specific data set allowed us to analyze additional haplochromine taxa not included in the GWA study outlined above. For these calculations, all mbuna (sans *A. labrosus*) sampled in this data set were treated as a single taxon, and all non-mbuna taxa possessing hypertrophied lips were grouped as another taxon (supplementary table S7, Supplementary Material online). Statistics (*D*, *f*_d_, and *f*_dM_) were then estimated for a trio consisting of the mbuna, *A. labrosu*s, and the non-mbuna species with hypertrophied lips. *Astatotilapia bloyeti* was set as the outgroup. We focused on the resulting *f*_dM_ scores (Malinsky et al. 2018 ), a modified version of the *f*_d_ statistic that was devised by Martin et al. (2015) to detect introgressed loci. These *f*_dM_ scores range from −1 to 1 and are distributed around zero, which is equivalent to no introgression. We quantified the proportion of gene flow between the hypertrophied lip non-mbuna and *A. labrosu*s (positive values) as well as the hypertrophied lip non-mbuna and the mbuna (sans *A. labrosu*s) (negative values) following Malinsky et al. (2021).

### Phylogenetic Topology Weighting

In order to use another tool to investigate allele sharing, we used the topology-weighting method TWISST v0.2 to examine the extent of phylogenetic discordance of loci across the genome (Martin and Van Bellegham 2017). Importantly, TWISST does not explicitly try to identify the causes of phylogenetic discordance. Phylogenetic discordance and subsequent elevated support for topologies conflicting with that of the species tree could result from either ancestral polymorphism (incomplete lineage sorting) or ancestral introgression. However, TWISST provides an independent method to further evaluate the Dinvestigate results although also allowing us to search for additional signals of possible introgressive hybridization between mbuna and non-mbuna taxa with hypertrophied lips. TWISST works by scoring all possible topologies for user-defined taxa set at different positions across the genome by determining the number of unique subtrees (i.e., where only one individual is sampled from each clade of interest) that corresponds to each of the alternative topologies. We focused on a subset of taxa consisting of 34 samples in total representing lineage both possessing hypertrophied lips as well as their small-lipped close relatives from the master SNP data set of Masonick et al. (2022) (supplementary table S8, Supplementary Material online). In TWISST, species were assigned to four clades: 1) non-mbuna haplochromines with hypertrophied lips (*C. euchilus*, *P. milomo*, *Placidochromis ornatus*, and *Placidochromis* “mbenji lip”), 2) one mbuna species with hypertrophied lips (*A. labrosus*), 3) all other sampled mbuna, and 4) the non-Malawi cichlid *Haplochromis bloyeti* used as an outgroup. Whereas *A. labrosu*s is clearly nested among the mbuna based on previous phylogenomic reconstructions (Masonick et al. 2022), we treated it here as a separate taxon to identify genomic regions that have potentially introgressed with that of hypertrophied lip non-mbuna through hybridization.

To infer relationships of genomic regions among the four delineated taxa above, we used scripts provided on Simon Martin's GitHub page (https://github.com/simonhmartin) and followed the general TWISST pipeline outlined therein to perform this four-clade TWISST. Briefly, the 1,012,749 biallelic SNPs specific to this taxonomic subset were phased with BEAGLE v5.1 and then prepared for subsequent analysis with the python script parseVCF.py. Maximum likelihood trees were estimated with RAxML v8.2.12 (Stamatakis 2014) under the GTRCAT model for windows of 250 nonoverlapping SNPs (×4,039 windows in total) using the script raxml_sliding_windows.py. Topology weighting was calculated considering all subtrees (–method complete) with the script twisst.py and the results were then visualized in RStudio using loess smoothing (span = 500 kbp) with the script plot_twisst.R (available at: https://github.com/simonhmartin/genomics_general/).

### Genomic Convergence with Other Vertebrate Phenotypes

For genomic regions showing genomic associations with the hypertrophied lip phenotype in the African Lake Malawi non-mbuna, we further assessed whether these regions contained genes previously identified in the literature as potentially underlying aberrant lip phenotypes in both cichlids and humans. First, we identified all the genes that were found in the 100-kbp regions centered around SNPs showing exceptionally high GWAs (supplementary tables S2 and S5, Supplementary Material online). Next, we compared these genes with four other types of genomic studies on vertebrate lips. We then contrasted our results to the homologous genes implicated through GWA mapping in dictating lip size in Central American Midas cichlids (Kautt et al. 2020) and compared the results with transcriptomic studies focused on gene expression in cichlids with hypertrophied lips. We also determined where our GWA study identified SNPs fell with respect to a QTL study examining lip area in hypertrophied lip cichlids endemic to Lake Victoria in Africa. Finally, for the genes in the 100 kbp-associated regions in the Malawi cichlids, we performed Google Scholar searches of the mammalian homolog gene names with the additional search terms “cleft lip”, “lip”, and “birth defect” to identify if any genes in our 100-kbp regions have been associated with congenital conditions affecting human lip phenotypes.

## Supplementary Material

evad072_Supplementary_Data

## Data Availability

Raw resequencing reads for the 15 sequenced Malawi cichlid genomes have been deposited in NCBI's Sequence Read Archive (see supplementarytable S1, Supplementary Material online).
