# Supplementary material for: A Kiss of Deep Homology: Partial Convergence in the Genomic Basis of Hypertrophied Lips in Cichlid Fish and Human Cleft Lip
Source: Genome Biol Evol. 2023 May 4;15(5):evad072. doi: 10.1093/gbe/evad072 (PMC10195091; doi:10.1093/gbe/evad072)
Supplement: evad072_Supplementary_Data [file evad072_supplementary_data.zip › Masonick_et_al_Supplementary_Material.docx]

**Supplementary Material**


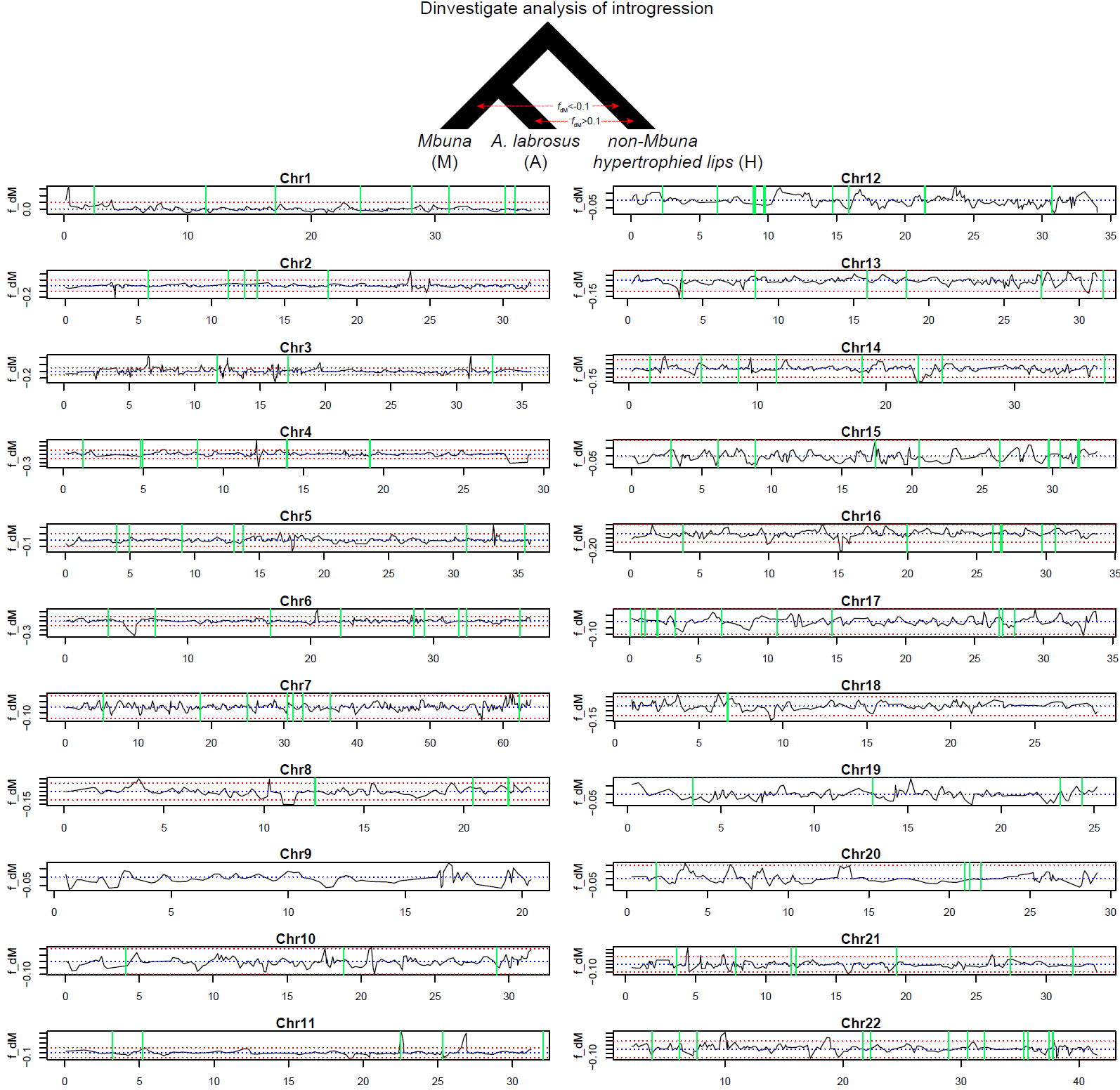


Supplementary Figure 1.

Supplementary Figure 1. Dinvesitgate *f*_dM_ scores calculated across the genome using sliding windows of 250 SNPs. The blue line denotes no signal of introgression (*f*_dM_ = 0.0). Values strongly departing from 0.0, rising above 0.1 or below -0.1 (red dashed lines), are taken here as evidence of putative introgression between either *A. labrosus* and the non-mbuna hypertrophied lip species or the mbuna (sans *A. labrosus*) and the non-mbuna hypertrophied lip species, respectively. Green bars denote the relative locations of the 233 significant SNPs showing strong association with the hypertrophied lip phenotype.


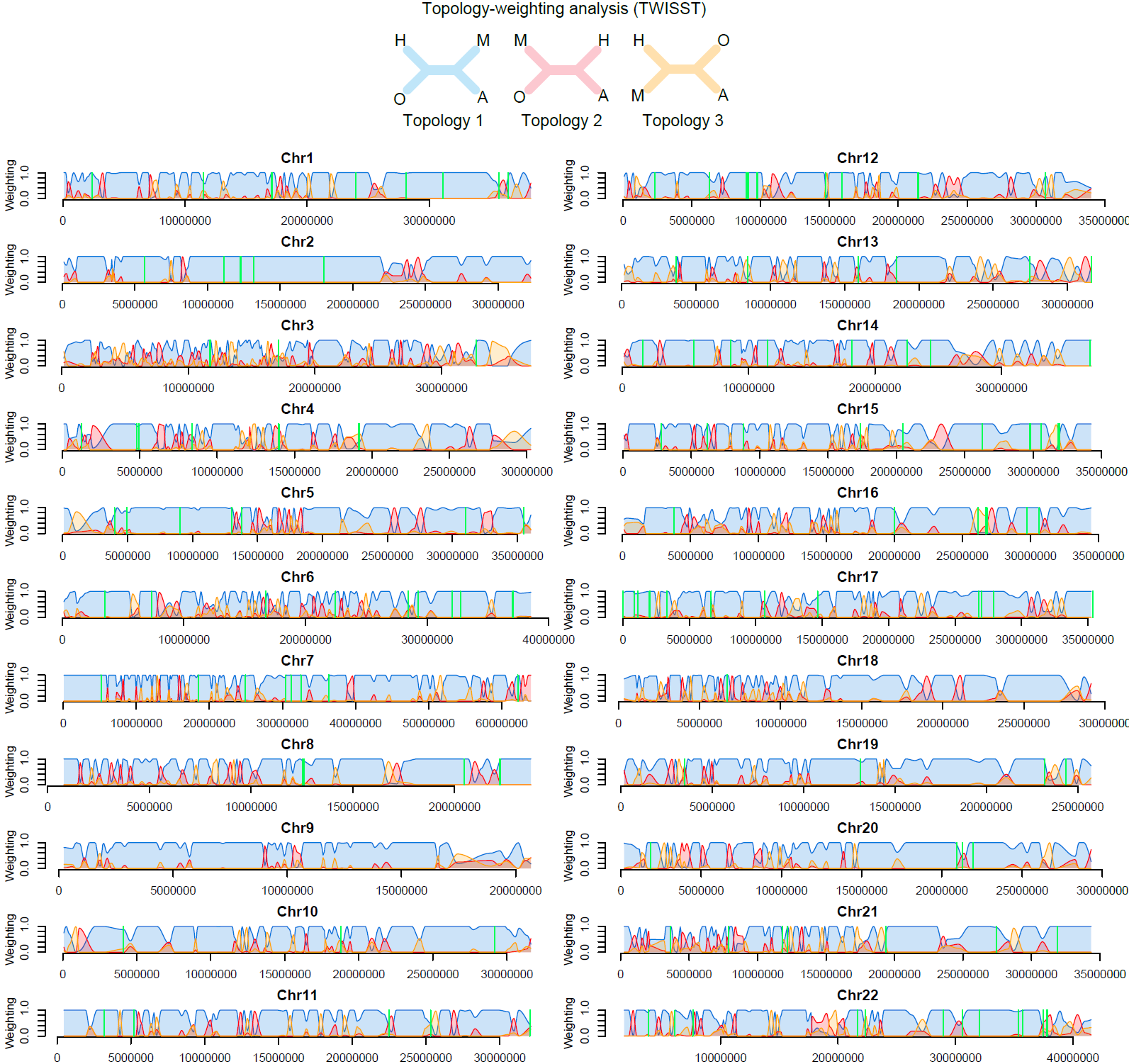


Supplementary Figure 2.

Supplementary Figure 2. TWISST topology frequencies estimated across the genome. This analysis was conducted to investigate whether the hypertrophied lip mbuna and non-mbuna were more likely to be monophyletic at putative regions of the genome than expected by chance. Blue represents the relative frequency of the species tree (i.e., the mbuna (M) and *A. labrosus* (A) are monophyletic), red peaks suggest a shared phylogenetic signal between *A. labrosus* and non-mbuna haplochromines with hypertrophied lips (H), and yellow peaks suggest a shared history between the mbuna (sans *A. labrosus*) and the non-mbuna haplochromines with hypertrophied lips. *Haplochromis bloyeti*, a species not native to Lake Malawi, was used as an outgroup. The frequency (weighting) of each topology was estimated across non-overlapping windows consisting of 250 SNPs and plotted with loess smoothing (span = 500-Kbp). Strong weightings for topologies other than that of the species tree could be the product of either ancestral polymorphism or genomic introgression. Green bars denote the relative locations of the 233 significant SNPs showing strong association with the hypertrophied lip phenotype.

***The following supplementary tables are provided in an Excel workbook:

Supplementary Table 1. Accession numbers for samples used in GWAS.

Supplementary Table 2. SNPs showing the highest-association (i.e., those surpassing the genome-wide P-value threshold of 5e-8) with the hypertrophied lip trait based on the GWA mapping analysis. For each SNP, the highest weighted topology from the TWISST analysis is indicated (T1, T2, or T2) as well as the Dinvestigate scores estimated from windows overlapping the SNP (significant scores, >0.1 or <-0.1, denoted in red).

Supplementary Table 3a. Dinvestigate statistics from all windows.

Supplementary Table 3b. Dinvestigate statistics of windows with significant SNPs only.

Supplementary Table 4a. TWISST topology weighting scores from all 4,039 windows.

Supplementary Table 4b. Subset of TWISST topology weighting scores from windows bearing significant GWAS SNPs.

Supplementary Table 5. Genes located within 50-Kbp (upstream and downstream) of SNPs showing the highest-association (i.e., those surpassing the genome-wide P-value threshold of 5e-8) with the hypertrophied lip trait based on the GWA mapping analysis. The gene details listed here were obtained from the gff3 file corresponding to the Maylandia zebra reference genome (M_zebra_UMD2a).

Supplementary Table 6. The inferred linkage group for hypertrophied lips from Henning et al. 2017, genetic position, LG from Mzeb genome (M_zebra_UMD2a), and the sequence used to build the linkage map.

Supplementary Table 7. Taxon designations for the Dinvestigate analysis in Dsuite. Pairs tested for introgression with Dinvestigate are highlighted. The specific trio we test here consisted of the "Mbuna_sister_group”, "Abactochromis_labrosus", and the "Utaka_fatlips".

Supplementary Table 8. Taxa used in the TWISST analysis and their clade designation (A = *Abactochromis labrosus*, M = mbuna sans *A. labrosu*s, H = hypertrophied lip non-mbuna taxa, and O = outgroup).
